# A Retrospective Analysis of Complaints to RSPCA Queensland, Australia, about Dog Welfare

**DOI:** 10.3390/ani9050282

**Published:** 2019-05-27

**Authors:** Hao Yu Shih, Mandy B. A. Paterson, Clive J. C. Phillips

**Affiliations:** 1 Centre for Animal Welfare and Ethics, University of Queensland, White House Building (8134), Gatton Campus, Gatton, QLD 4343, Australia; c.phillips@uq.edu.au; 2 Royal Society for the Prevention of Cruelty to Animals Queensland, Brisbane, QLD 4076, Australia; mpaterson@rspcaqld.org.au

**Keywords:** dog, canine welfare, canine cruelty, neglect, RSPCA, age

## Abstract

**Simple Summary:**

Animal neglect and cruelty are important welfare and social issues, and dogs are one of the most commonly reported species to have experienced both. Most previous studies related to canine cruelty and welfare focused on animal abuse and dog fighting. However, literature dealing with the milder but more common forms of animal welfare concerns is limited. Therefore, this retrospective study aimed to understand the epidemiology of different types of canine welfare complaints in Queensland in the past decade and also to identify risk factors and their roles in different types of welfare complaints. The number of complaints received each year increased by 6.2% annually. The majority of complaints were neglect-related rather than related to deliberate cruelty, with the most common complaints being that dogs had poor body conformation, insufficient food and/or water, and receiving inadequate exercise. Poor living conditions and leaving dogs in a hot vehicle unattended were more commonly reported in recent years, potentially due to higher public awareness. Adult dogs that were reported were more likely to be alleged to have been poisoned, left unattended in a hot car, abandoned, and to have had inadequate exercise and shelter, compared with puppies. Puppies that were reported were more likely to be alleged to have experienced cruelty, lack of veterinary support, overcrowding, poor living and health conditions, and inappropriate surgery. Recognising which dogs are at most risk of cruelty will inform strategies to address this serious welfare problem.

**Abstract:**

Animal neglect and cruelty are important welfare and social issues. We conducted an epidemiological study of dog welfare complaints and identified risk factors. The retrospective study included 107,597 dog welfare complaints received by RSPCA Queensland from July 2008 to June 2018. The risk factors considered were the age of dogs and the year of being reported. The number of complaints received each year increased by 6.2% per year. The most common complaints were poor dog body conformation, insufficient food and/or water, dogs receiving inadequate exercise, and dogs being confined or tethered. Increasing numbers were most evident for poor living conditions and leaving dogs in a hot vehicle unattended, both of which may have resulted from increasing public awareness. The majority of complaints were neglect-related rather than related to deliberate cruelty. Compared with puppies, adult dogs were more likely to be reported to have been poisoned, left unattended in a hot car or abandoned, as well as to have had inadequate exercise and shelter. Reported puppies were more likely to be alleged to have experienced cruelty, lack of veterinary support, overcrowding, poor living and health conditions, and inappropriate surgery. In conclusion, animal neglect was the most commonly reported welfare concern in dogs. Due to an assumed increasing public awareness of some types of cruelty, the trends of reported concerns differed. Adult dogs and puppies were reported to be involved in different types of welfare concerns. Strategies to address cruelty to dogs can be informed by an understanding of risk factors and trends in types of cruelty.

## 1. Introduction

Animal cruelty involves all human behaviours towards animals that are morally and/or legally unacceptable, causing them to be inflicted with unnecessary and unjustifiable physiological, psychological, and behavioural discomfort or pain [1,2]. It is a complex issue implicating animal welfare, moral concerns, criminal activity, and violence [2,3,4]. It is regulated by state and territory law in Australia; for example, in Queensland by the Animal Care and Protection Act 2001 (ACPA [1]). This state-based legislation empowers the State to appoint inspectors, some of whom are employed by the Royal Society for the Prevention of Cruelty to Animals, Queensland (RSPCA Qld), to investigate potential breaches of the Act and enforce compliance with the Act [1]. There are two main offences under the ACPA, one is failure to fulfil duty of care responsibilities and the other is cruelty. There are a number of other specified offences. The Act recognises that a person who has charge of an animal owes that animal a duty of care. Failure to provide such care potentially constitutes a ‘breach of duty of care’ offence. This offence covers such actions as not providing sufficient food, water, exercise, veterinary care, and suitable living conditions. It is not only the owner that has a duty of care towards an animal. Anyone who even temporarily is in charge of an animal has a duty of care. The second major offence is ‘animal cruelty’ and the Act describes what it sees as cruelty in Section 18. A cruel act towards an animal can be committed by anyone, whether it is their own animal, another domestic animal, or even a wild animal [1]. It is important to note, that under the ACPA, the intention of a person to be cruel is not a prerequisite for committing the offence of cruelty. If an action carried out by a person causes pain and suffering and the action was intentional (that is not accidental), the person may be charged with cruelty. The intention to carry out the action must be proved but not the intention to be cruel. If a lack of action deprives an animal of its fundamental needs then they may be charged with a breach of their duty of care or even cruelty, depending on the circumstances. Motivation may be considered during sentencing [1]. Other offences under the Act include unreasonable abandonment or release, the carrying out of prohibited surgical procedures (e.g., tail docking, ear cropping, debarking, etc.), being involved in, or having items used for, a prohibited event such as dog or cock fighting, and allowing an animal to injure or kill another animal [1].

Potential cases are reported to RSPCA through various means. RSPCA Qld has a ‘Cruelty Complaints’ telephone number manned 24 h a day, seven days a week; complaints also come in through emails. Complaints can be made by members of the public but also by veterinarians and veterinary nurses, council officers, and other government and non-government employees visiting a location as part of their duties. Animals surrendered to the RSPCA or that come in as strays may be investigated if cruelty is suspected. They are considered by RSPCA Qld inspectors and further investigated if necessary.

According to the annual statistics of RSPCA Qld, there were 15,102 animal welfare complaints reported by the general public in 2011 [5], which had increased to 17,929 by 2017 [6]. Of all species falling victim to animal welfare concerns, dogs (*Canis familiaris*) are one of the most commonly reported species [7].

Various risk factors have been identified as contributing to an unsuccessful dog–owner relationship, which potentially results in neglect or abuse. These include the age of the dog [8,9], dog behaviour [8,10,11,12], physical attributes of the dog [9,13], the owner’s motivation to care for the dog [14,15], the owner’s attachment to the dog [12,16], costs of keeping the dog [16,17], and the owner’s socioeconomic status [18,19]. In relation to actions carried out by third parties, most studies have focused on organised industries such as dog coursing [20] and fighting [21]. There has also been research into the origin of ‘noxious abuses’, e.g., cruelty involving intentional abuse, such as beating, shooting, and burning, that lead to severe physical injuries to the animals [7,15]. Literature dealing with the milder but more common forms of animal welfare concerns is limited. One report considers neglect, such as exposing dogs to poor nutrition, keeping dogs in a backyard for hours without a shelter, and failing to meet exercise needs [2]. Most studies [20,22,23] stress the moral, legal, and social aspects of animal cruelty, and few explore the epidemiological dimension of this topic. This study addresses the epidemiology of diverse animal welfare concerns reported by the general public, instead of actual neglect or cruelty cases in a typical Western society. It also aims to identify the age of dogs as a risk factor for different forms of canine welfare complaints. Other risk factors, breed and socioeconomic status of the complainant, will be the subject of future papers.

## 2. Materials and Methods

### 2.1. Materials

From July 2008 to June 2018, RSPCA Qld received 129,036 canine welfare complaints. Some involving more than one dog were recorded as multiple complaints sharing the same case number, while others were recorded as one complaint with multiple animals. To avoid sample bias due to multiple entries, we only retained the first complaint of case numbers with multiple entries, discarding 21,439 entries as a result. There remained 107,597 canine welfare complaints for this retrospective study. Complaints that fell within the zone of responsibility of RSPCA Qld (determined by a Memorandum of Understanding between RSPCA Qld and Biosecurity Queensland, the Government Department tasked with the administration of ACPA) were investigated by RSPCA Qld inspectors. All other complaints were referred to Biosecurity Queensland to be investigated by their inspectors.

Complaints were recorded in Shelter Buddy^®^, the RSPCA Qld database. The following information was requested from the reporter of each incident by the inspector at the time of taking the complaint: the number of dogs involved (n = 106,104), their age (n = 107,597), their breed(s) (n = 92,021), the coded complaint type(s) (n = 106,983), the suburb (n = 107,413), and the postcode (n = 107,270); in addition, the date was recorded (n = 107,597). Dogs’ ages were dichotomised into adult dog and puppy, based on reporters’ interpretation. It is important to recognise that the information recorded from the complainant may have been inaccurate or inaccurately interpreted, e.g., a small dog is commonly referred to as a puppy. Records regarding breed and the number of dogs involved were based on either complainants’ initial reports or comments from trained inspectors, again recognising inaccuracies with identification of the breed and the number of dogs involved. The ‘complaint code’ was selected by the staff member receiving the call or email from a drop-down menu of 18 possible complaints (Appendix A, Table A1). Multiple ‘complaint codes’ were able to be selected for each case, according to the description of what was alleged to have happened to the dog(s), and each was treated as a separate code for analysis.

### 2.2. Statistical Analysis

Data were analysed using the statistical package Minitab^®^ 17.3.1. Descriptive analysis was used to investigate the distribution of complaint codes. Polynomial regression analysis and simple linear regression analyses were used to model the prevalence of different complaint codes from 2008 to 2018. The model chosen was that with the highest R-sq value, after ensuring that all components in the model were significant (*p* < 0.05). In 2008 and 2018, only data from July to December, and January to June were available, respectively. Therefore, data in 2009 and 2017 were used to test for within year variation in code citation rates for 2008 and 2018, respectively. Specifically, chi-squared analyses were conducted to compare whether the reported prevalence of each complaint code from January to June were different from those in July to December in 2009 and 2017. If there was no significant (*p* < 0.05) difference between the two six-month periods in that complaint code in 2009 and/or 2017, then the prevalence of the particular complaint code in the six-month period in 2008 and/or 2018 was/were assumed to be partially representative of the entire year(s). However, if there was a significant difference between the two six-month periods in that complaint code in 2009 and/or 2017, the data of the specific complaint code in 2008 and/or 2018 were excluded from the polynomial regression analyses of year effects. After that, a Grubbs’ test was used to identify outliers of each complaint code, which were excluded from polynomial and simple linear regression analyses. In polynomial regression analyses and simple linear regression analyses, years were entered as input variables and the prevalence of the complaint code as the output. The models were chosen on the basis of significant *p* values and the greatest R-sq values yielded. Three complaint codes, Causing captive animal to be injured/killed by a dog (N = 29), Keeping or using animal for blooding/coursing a dog (N = 18), and Emergency relief (N = 8) were not included in polynomial and simple linear regression analyses because the number of reported cases in the past decade was too few. Eighteen stepwise forward binary logistic regression models were constructed to understand how dogs’ ages correlated with each complaint code. To determine the effect of age on complaint codes, age was entered (in dichotomous data form) into a binary logistic regression model as a fixed factor, using a logit function, with an alpha value to enter of 0.15. Complaint codes were entered into the model as outcomes. Separate models were constructed for each complaint code with the same input variable.

## 3. Results

### 3.1. Complaint Codes and Dogs’ Ages

**Figure 1:**
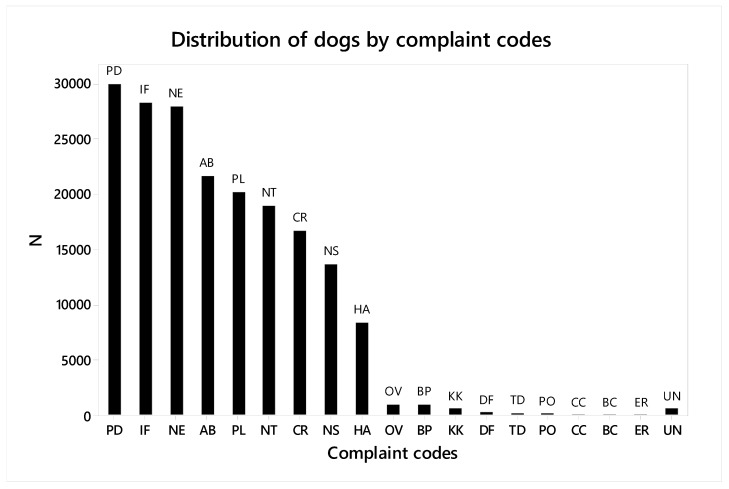
Distribution of dogs by complaint code. PD-Poor dog condition (27.9%, N = 29,982); IF-Insufficient food and/or water (26.3%, N = 28,265); NE-No exercise/confined/tethered (25.9%, N = 27,913); AB-Abandonment (20.1%, N = 21,626); PL-Poor living condition (18.7%, N = 20,162); NT-No treatment (17.6%, N = 18,963); CR-Cruelty (15.5%, N = 16,661); NS-No shelter (12.7%, N = 13,682); HA-Hot animal in car (7.8%, N = 8384); OV-Overcrowding (0.9%, N = 978); BP-Baiting/poisoning (0.9%, N = 974); KK-Knowingly allowing an animal to kill/injure another (0.6%, N = 600); DF-Dog fighting or other prohibited offence (0.3%, N = 277); TD-Tail docking or other surgical procedure (0.2%, N = 214); PO-Prohibition order breached (0.1%, N = 133); CC-Causing captive animal to be injured/killed by a dog (0.03%, N = 29); BC-Keeping or using animal for blooding/coursing a dog (0.02%, N = 18); ER ^[a]^-Emergency relief (0.01%, N = 8); UN-Unknown (0.6%, N = 614). ^[a]^ Emergency relief, as opposed to emergency rescuing which occurred when an animal encountered an urgent situation not related to domestic violence, was provided based on the ACPA, Section 123 [1].

There were 18 complaint codes in total (Appendix A Table A1). On average, each case involved 1.76 (SEM = 0.003) codes. The distribution of complaint codes is presented in Figure 1. The most common codes, listed in declining order, were Poor dog condition (n = 29,982, 27.9%), Insufficient food and/or water (n = 28,265, 26.3%), No exercise/confined/tethered (n = 27,913, 25.9%), and Abandonment (n = 21,626, 20.1%). Overall, 93.67% (N = 100,791) of reported cases involved reported adult dogs and 6.33% (N = 6806) of reported cases involved reported puppies.

### 3.2. Trends of Complaint Types

The number of complaints received annually increased by 6.2% per year, and the incidence of most complaint codes changed over the ten years. Results of the Chi-squared analyses showed that the prevalence from January to June and from July to December was significantly (*p* < 0.05) different for Poor living condition and Baiting/poisoning in 2009, No treatment and Poor dog condition in 2017, and No exercise/confined/tethered and Hot animal in car in both 2009 and 2017 (Appendix A, Table A2). Therefore, the data for Poor living condition and Baiting/poisoning in 2008, No treatment and Poor dog condition in 2018, and No exercise/confined/tethered and Hot animal in car in both 2008 and 2018 were excluded from the analyses of year effects. The prevalence of Poor dog condition in 2008 was an outlier (*p* = 0.029), and therefore was excluded as well. Figure 2 demonstrates the trends and the equations used for polynomial regression or simple linear analysis of each complaint code. These trends can be classified into five patterns: negative linear, positive linear, concave, monotonic, and irregular. Negative linear models included No exercise/confined/tethered, overcrowding, and Tail docking or other surgical procedure. Positive linear models included Poor living conditions, Hot animal in car, and Prohibition order breached. A concave pattern, indicating that the prevalence increased to a peak and then slowly decreased, was observed for No treatment, Abandonment, No shelter, and Knowingly allowing an animal to kill/injure another, for which codes the prevalence reached a peak in 2015, 2014, 2015, and 2011, respectively. In monotonic patterns, the trend was to generally increase, but not consistently, e.g., the prevalence of Poor dog condition generally increased, except for 2011–2016. Finally, some complaint codes had irregular changes over time. Cruelty, Insufficient food and/or water, Baiting/poisoning, and Dog fighting or other prohibited offence could not be modelled as they were reported sporadically over the ten years.

### 3.3. Risk Factors for Different Complaint Codes

**Figure 3:**
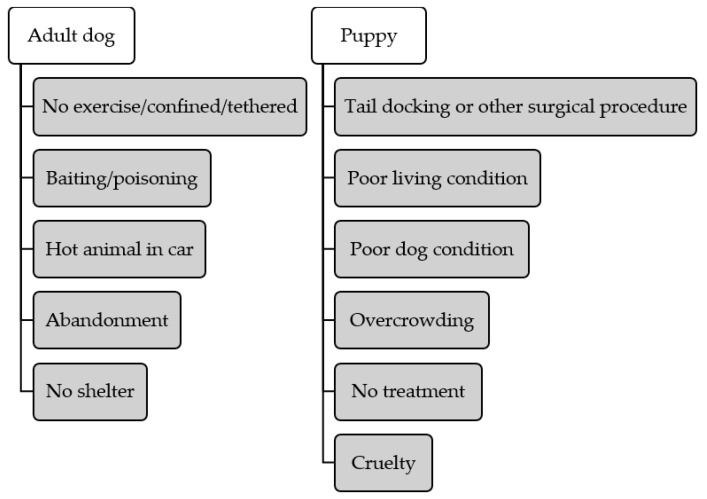
Positive relationships between dog age (adult dog and puppy) and complaint codes. Complaint codes listed under ‘Adult dog’ and ‘Puppy’ are complaints commonly involving adult dogs and puppies, respectively.

We considered age as a risk factor. The relationships between dogs’ age and complaint codes are displayed in Table 1 and Figure 3. Compared to adult dogs, puppies were more likely to be reported for alleged Tail docking or other surgical procedure (OR = 9.87, *p* < 0.001), Overcrowding (OR = 4.44, *p* < 0.001), Poor living condition (OR = 1.45, *p* < 0.001), No treatment (OR = 1.33, *p* < 0.001), Cruelty (OR = 1.27, *p* = 0.001), and Poor dog condition (OR = 1.23, *p* < 0.001). Adult dogs were significantly more likely to be reported as an alleged case of a Hot dog in car (OR = 0.41, *p* < 0.001), Baiting/poisoning (OR = 0.42, *p* < 0.001), Abandonment (OR = 0.53, *p* < 0.001), No exercise/confined/tethered (OR = 0.64, *p* < 0.001) and No shelter (OR = 0.91, *p* = 0.037).

## 4. Discussion

### 4.1. Complaint Codes

This study reports the prevalence and progression of canine welfare complaints over the past decade. The complaints came from members of the public and may not represent all animal welfare issues or breaches of the ACPA. It must also be recognized that not all calls were found, on investigation, to represent a breach of the ACPA or even a dog suffering poor welfare. The number of complaints may be a representation of the degree of awareness in the community of animal welfare and the vigilance of many. A small number may be vexatious.

The descriptive analyses of different complaints show that the majority of alleged complaints related to neglect rather than deliberate maleficence. In this study, poor body and living conditions, insufficient food and water, and lack of the provision of exercise were the most commonly reported. This is in line with previous research that animal neglect or cruelty was most likely a result of ignorance due to lack of knowledge or forgetfulness [2,4].

In most cases, but particularly where it was decided not to continue to a prosecution, education of the dog owner was undertaken. To address neglect-related issues, people in charge of the animal were informed about food and water requirements, as well as the need for exercise, human companionship, and what represents good living conditions for a dog. For example, diets were recommended that are complete and balanced to replace homemade and all raw meat diets [24], and dog owners were likely to be informed that there were specialized products designed for specific ages of dogs [25] or dogs with specific health concerns [26]. Regular exercise is essential to promote health and quality of life [13,27,28]. Unfortunately, there are sometimes mismatches between an owner’s exercise capability and the needs of their dog which inspectors recognise and advise accordingly [13,28]. The amount and frequency of exercise recommended varies according to the age, breed, size, fitness, and health of the dog. According to the ACPA, owners should ensure their dogs exercise for two hours after being continuously confined (e.g., caged or tethered) for 24 h, or for 1 h after 24 h confinement and another 1 h in the next 24 h [1]. This is the minimal time for most dogs to exercise and the majority benefit from receiving more [29]. The United Kingdom kennel club has published guidelines detailing suitable exercise amounts for each breed [29]. These are general in nature and not prescriptive.

Poor living conditions are negatively associated with a dog’s quality of life, and increase the risk of diseases caused by ringworm, Giardia, Cryptosporidium, Toxocara, and Ancylostoma, as well as infestation with ectoparasites such as fleas [30,31,32,33]. Some of these agents are zoonotic and can cause health problems in humans.

### 4.2. Trends Over Time

Overall, the number of complaints received each year increased by 6.2% per year in the past decade, which may be contributed by the increasing population in Queensland in parallel with growing dog ownership in Queensland, and people’s rising awareness of animal welfare. The population growth rate in Queensland was around 2% per year from 2008 to 2017 [34,35], and the pet population breakdown across the states and territories mirrored the country’s population distribution [36]; it is reasonable that dog ownership in Queensland has also been increasing at a similar rate. Given the mismatch between the growth rate of canine welfare complaints (around 6% annually) and Queensland population (around 2% annually) for the same period of time, we believe that increasing public awareness and propensity to report animal cruelty may be another contributing factor [37]. The issue of animal welfare is becoming popular with the Australian public, in terms of their knowledge, concerns, willingness to participate, and legislation [38,39]. This is reflected by the reported increased concern for animal welfare among the general public [40,41,42], animal protection movements from advocacy groups [43], and public media [44], as well as the development of a closer relationship between humans and animals [45] and people’s inclination to report cases involving animal harm to the police and RSPCA [37]. Such an increase in public awareness may be associated with the connections between animal abuse and human violence [23,46], and animal and human health [30,47], as well as more general dog ethical issues, such as dog consumption in Asia [48]. Moreover, people increasingly acknowledge the importance of good nutrition [49], canine behaviour [50,51] and emotions [52], and the goal of achieving a no-kill policy and improving the live release rate for roaming or sheltering dogs [39,53]. At RSPCA Qld, the number of volunteers has also grown from 2000 in 2011 to over 5000 in 2018 [6], suggesting that more people are actively concerned about the welfare of dogs.

The trends seen with the different complaints are also important to consider. Dogs were commonly reported as being in poor condition, living in poor environments, or being left in a heated vehicle. The increasing frequency of these three complaints may also be the result of a growing awareness of animal welfare among the public. However, the importance of animal welfare law enforcement and public education should not be underestimated. Humans’ changing lifestyle may also influence the prevalence of certain kinds of animal welfare concerns. For instance, more people nowadays own a car and travel with their dogs frequently [54]. Consequently, dogs are at greater risk of being left unattended in a vehicle. This is particularly hazardous in Queensland, where median summer maximum temperatures of around 30 °C can lead to dangerously high temperatures inside cars [55]. Apart from the increasing trends for the types of complaints mentioned above, some complaint categories, particularly those involving food or water insufficiency, cruelty, dog poisoning, and dog fighting demonstrated irregular patterns, suggesting that they have occurred, or have been reported, inconsistently over the ten years [56]. These complaints also have the potential to jeopardize the dogs’ life, and may also be related to violence and crimes [4,21,57]; therefore, these should be closely monitored. Finally, in Queensland, tail docking and other inappropriate surgical procedures (e.g., ear cropping and declawing) have been banned under the ACPA since 2001, unless they are undertaken by and under the recommendation of a veterinary surgeon [1]. Our results demonstrate a steady decline in the number of such cases, which indicates that the law is being adhered to.

### 4.3. Adult Dogs and Puppies

Apart from gaining an overall understanding of the prevalence and trends of different complaints, we also identified dog age as a risk factor in our dataset. Figure 3 summarizes the positively significant correlations between dog age and complaint codes. Adult dogs were more likely to be alleged to be subjected to activity-related welfare issues—for example being confined. They were also more likely to be reported as abandoned or left in a heated vehicle. The higher abandonment potential is supported by an anthropomorphic theory that states that as adult dogs possess fewer infant-like traits, and thus are regarded as less attractive, they are less likely to evoke our nurturing instinct, creating a weaker bond with humans [12,58,59]. Adult dogs may have a greater chance of being left in a hot car because they are more difficult to carry and are usually not allowed to enter public places. On the other hand, puppies need more care and are easier to carry with a portable confinement box, therefore, it is less likely that they will be transported in a car and left when the driver is absent. Drivers may also imagine adult dogs to be more robust than puppies and more able to cope with high temperatures. This problem may be solved by promoting more pet-friendly environments (e.g., shops and restaurants) where dogs can stay with their owners and not be left in a vehicle alone. Increased information and signage in car parks reminding drivers of the danger to their dog of hot cars should also be encouraged. As for puppies, tail docking is generally conducted on three to five-day old puppies [60,61]; therefore, it is not surprising that this complaint category was more commonly reported with puppies than with adults. As the ban on tail docking was introduced in Queensland in 2001 [1], it is likely that some adult dogs were not reported because they were docked before the ban was introduced. 

Additionally, in this study, puppies were alleged to be more likely to suffer from cruelty, which may result from the fact that puppies are submissive, and therefore tend to satisfy the controlling motivation for animal cruelty [15]. This finding is partially supported by a previous study that focused on non-accidental injuries of dogs and cats, and which revealed that dogs less than two years of age were more susceptible to intentional abuse [62]. In addition to the submissive nature of puppies, the authors suggest that young dogs are less manageable and thus may provoke owners with aggressive potential [62]. Another inconclusive result was found in a research trial investigating animal cruelty and domestic violence; in that study, authors did not find dogs’ age to be a predisposing factor of being targeted for abuse [46]. Finally, access to appropriate medical support and suitable living conditions such as a good environment and enough space is another concern for puppies. These welfare concerns are probably indicators for animal hoarding or puppy farms [63,64].

### 4.4. Strengths and Limitations

One strength of this study is that it describes the trends in dog welfare complaints in Queensland over the last ten years and represents a large database, which allowed trends to be determined. It also correlates dog age with specific complaints. However, the current study also has its limitations. First, dogs were only classified as puppy or dog by complaints, which may hide important details related to age [9]. Second, this dataset covers only coastal, highly populated parts of Queensland, and thus generalization should be made with caution. Finally, the complaint code choices made by Call Centre staff were made on the basis of information obtained from the public, which indicates the potential of inaccurate reports. However, the study presents an analysis of what was reported and reflects changes in public awareness and motivation to act. Future studies could assess the accuracy of what people report and include more risk factors; for instance, breeds, behavioural issues of dogs, socioeconomic levels, and the history of an unsuccessful ownership have been reported to negatively influence dog ownership [8]. Dealing with behavioural problems and preventing people with a history of poor dog ownership from acquiring a new dog could reduce the risk of similar incidents being repeated. These factors are useful for addressing canine welfare issues and associated crimes and thus should be considered in future studies.

## 5. Conclusions

This study identified prevalence, trends, and the age of dogs as a risks factor for different types of complaints. Breed of the dog and socioeconomic status of the complainant will be the subject of future papers. Some neglect-related complaints, such as offering insufficient food and water, providing poor living conditions, and leaving a dog unattended in a heated vehicle apparently became more prevalent in recent years, probably indicating greater public awareness rather than an increase in neglectful behaviour. However, some serious complaints have been consistently reported over the past decade, including those involving animal abuse or severe injuries, and consequently should be closely monitored. The age of dogs was correlated with complaints about abandonment, neglect-related mistreatment, cruelty, and inappropriate surgery. Adult dogs were more likely to be reported as receiving inadequate exercising and shelter, having been abandoned, and having been left unattended in a hot vehicle; puppies were more likely to be reported as having poor living and health conditions, having undergone inappropriate surgery, and having suffered abuse. Recognising which dogs are at most risk of cruelty will inform strategies to address this serious welfare problem. Furthermore, the local or state government can direct specific attention to the most common and growing types of neglect and cruelty.

## Figures and Tables

**Figure 2:** Polynomial regression of each complaint code. The X axis represents the year, and the Y axis represents the prevalence of the complaint code.

**Table 1:** Odds ratio of each variable in the logistic regression model of complaint codes. The outputs of these models were different complaint codes. The input variable was dog age (puppy or dog).

Complaint Code	Puppy/Dog OR (CI) ^(a)^	*p* Value
Tail docking or other surgical procedure	9.87 (7.30, 13.34)	<0.001
Overcrowding	4.44 (3.70, 5.32)	<0.001
Poor living condition	1.45 (1.35, 1.55)	<0.001
No treatment	1.33 (1.24, 1.42)	<0.001
Cruelty	1.27 (1.18, 1.37)	0.001
Poor dog condition	1.23 (1.16, 1.30)	<0.001
No shelter	0.91 (0.84, 1.00)	0.037
No exercise/confined/tethered	0.64 (0.60, 0.69)	<0.001
Abandonment	0.53 (0.49, 0.58)	<0.001
Baiting/poisoning	0.42 (0.27, 0.66)	<0.001
Hot animal in car	0.41 (0.35, 0.47)	<0.001
Causing captive animal to be injured/killed by dog	-- ^[b]^	-- ^[b]^
Dog fighting or other prohibited offence	-- ^[b]^	-- ^[b]^
Emergency relief	-- ^[b]^	-- ^[b]^
Insufficient food and/or water	-- ^[b]^	-- ^[b]^
Keeping or using animal for blooding/coursing a dog	-- ^[b]^	-- ^[b]^
Knowingly allowing an animal to kill/injure another	-- ^[b]^	-- ^[b]^
Prohibition order breached	-- ^[b]^	-- ^[b]^

^(a)^ Dog age was only classified as dog or puppy. Odds ratio refers to puppy relative to dog. ^(b)^ Age factor was not selected in the logistic regression model.

## Appendix A

**Table A1:** Description of each complaint code alleging a welfare issue.

Complaint Code	Description
Abandonment	An animal was abandoned/left by the owner either at their abode or somewhere else such as in the bush.
Baiting/Poisoning	An animal was poisoned or planned to be poisoned.
Causing captive animal to be injured/killed by dog	A person let a captive animal be injured/killed by a dog.
Cruelty	A person was reported to have abused an animal.
Dog fighting or other prohibited offence	A person was reported for allowing dogs to fight or conducting other specifically prohibited acts.
Emergency relief	Emergency relief is required for an animal left unattended because its owner experienced an emergency (e.g., flood or being hit by a car).
Hot animal in car	An animal was left unattended in a car during hot weather.
Insufficient food and/or water	An animal has insufficient food and/or water.
Keeping or using animal for blooding/coursing a dog	A person used a live bait for blooding/coursing a dog.
Knowingly allowing an animal to kill/injure another	A person allowed one animal to kill/injuring another one, and did nothing to stop them.
No exercise/confined/tethered	An animal is confined or tethered and not given a suitable amount of exercise.
No shelter	An animal is not provided with suitable shelter provisions.
No treatment	An animal did not receive appropriate medical treatment when needed.
Overcrowding	The number of animals is too high for the living space provided.
Poor dog condition	The general condition of an animal is poor. (e.g., messy/matted coat, pussy eyes, etc.)
Poor living condition	The living environment of the animal is poor.
Prohibition order breached	An owner violated a prohibition order ^(a)^.
Tail docking or other surgical procedure	Tail docking or other surgical procedure (e.g., declaw removal, etc.) was conducted on an animal.
Unknown	Unknown

^(a)^ Prohibition order—A prohibition order is given by the court when a person convicted of an animal welfare offense must not possess any or a specific animal for a prescribed period of time [1].

**Table A2:** Chi-squared tests comparing prevalence of different complaint codes in two six-month periods, from January to June and from July to December, in 2009 and 2017.

Complaint Code	Prevalence % (N) January–June, 2009	Prevalence % (N) July–December, 2009	*p*-Value	Prevalence % (N) January–June, 2017	Prevalence % (N) July–December, 2017	*p*-Value
No Treatment	13.2% (500/3787)	13.9% (576/4134)	0.343	18.9% (1203/6369)	17.1% (1135/6632)	0.008
Abandonment	13.8% (523/3787)	14.5% (601/4134)	0.354	20.4% (1298/6369)	19.4% (1288/6632)	0.171
Cruelty	15.2% (575/3787)	16.7% (692/4134)	0.059	14.6% (933/6369)	14.6% (970/6632)	0.970
Insufficient food and/or water	26.9% (1017/3787)	25.9% (1073/4134)	0.364	24.4% (1551/6369)	24.1% (1599/6632)	0.748
No shelter	11.3% (428/3787)	12.4% (513/4134)	0.128	13.2% (843/6369)	14.3% (948/6632)	0.080
No exercise/confined/tethered	31.8% (1206/3787)	29.7% (1226/4134)	0.035	21.4% (1366/6369)	23.4% (1554/6632)	0.007
Hot Animal in Car	4.7% (177/3787)	6.2% (256/4134)	0.003	8.3% (530/6369)	9.5% (630/6632)	0.018
Poor living conditions	13.8% (523/3787)	15.5% (640/4134)	0.036	23.0% (1462/6369)	22.5% (1490/6632)	0.507
Baiting/poisoning	0.7% (27/3787)	1.2% (51/4134)	0.018	0.8% (52/6369)	1.1% (73/6632)	0.097
Poor dog condition	24.1% (914/3787)	23.1% (956/4134)	0.291	31.9% (2033/6369)	29.4% (1948/6632)	0.002
Overcrowding	1.6% (61/3787)	1.8% (73/4134)	0.593	0.4% (25/6369)	0.3% (20/6632)	0.377
Knowingly allowing an animal to kill/injure another	0.5% (20/3787)	0.6% (23/4134)	0.864	0.3% (20/6369)	0.2% (14/6632)	0.250
Tail docking or other surgical procedure	0.3% (13/3787)	0.3% (12/4134)	0.675	0.1% (9/6369)	0.2% (11/6632)	0.721
Dog fighting or other prohibited offence	0.2% (6/3787)	0.2% (7/4134)	0.905	0.2% (10/6369)	0.3% (19/6632)	0.118
Prohibition order breached	0% (0/3787)	0% (0/4134)	--	0.2% (15/6369)	0.2% (10/6632)	0.270

-- Unable to conduct Chi-squared test because no cases were reported in 2009 and 2017.
